# Supplementary figures and images for: Rumen sampling methods bias bacterial communities observed
Source: PLoS One. 2022 May 5;17(5):e0258176. doi: 10.1371/journal.pone.0258176 (PMC9070869; doi:10.1371/journal.pone.0258176)

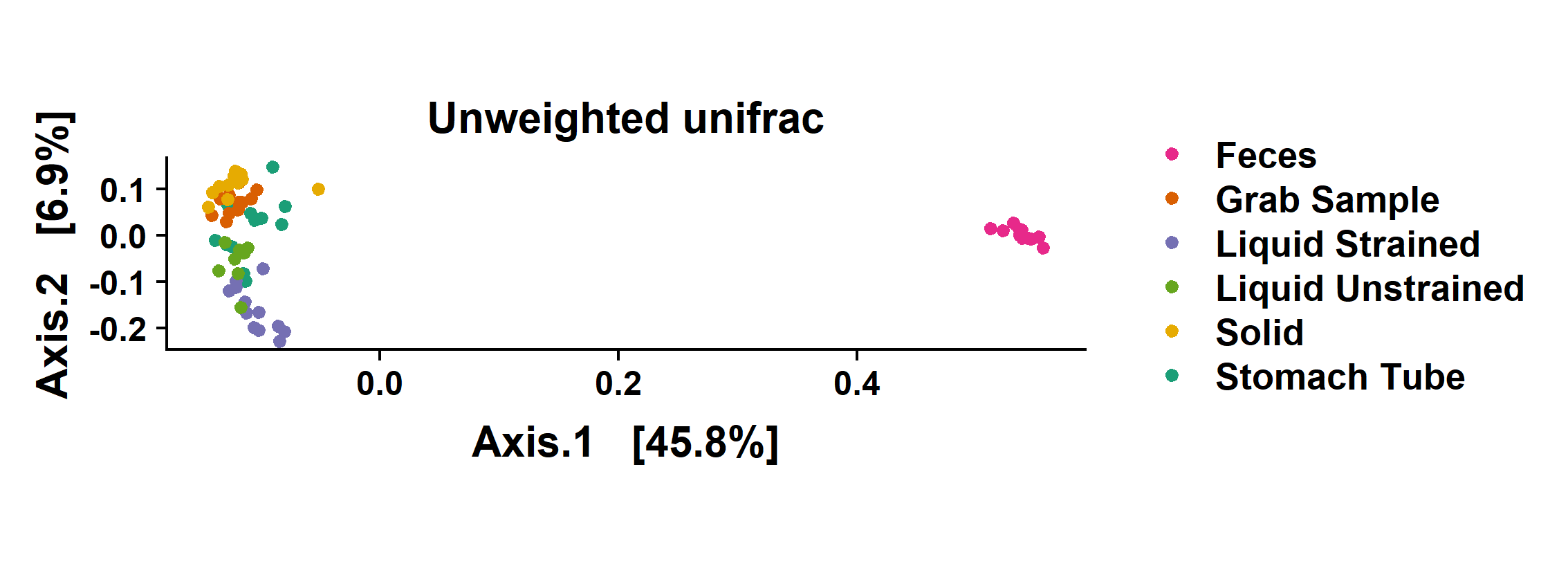

Supplement: S1 Fig — To faithfully reflect the variance in the coordinates, the height-to-width ratio was based on the ratio between the corresponding eigenvalues. (TIF) [file pone.0258176.s001.tif]

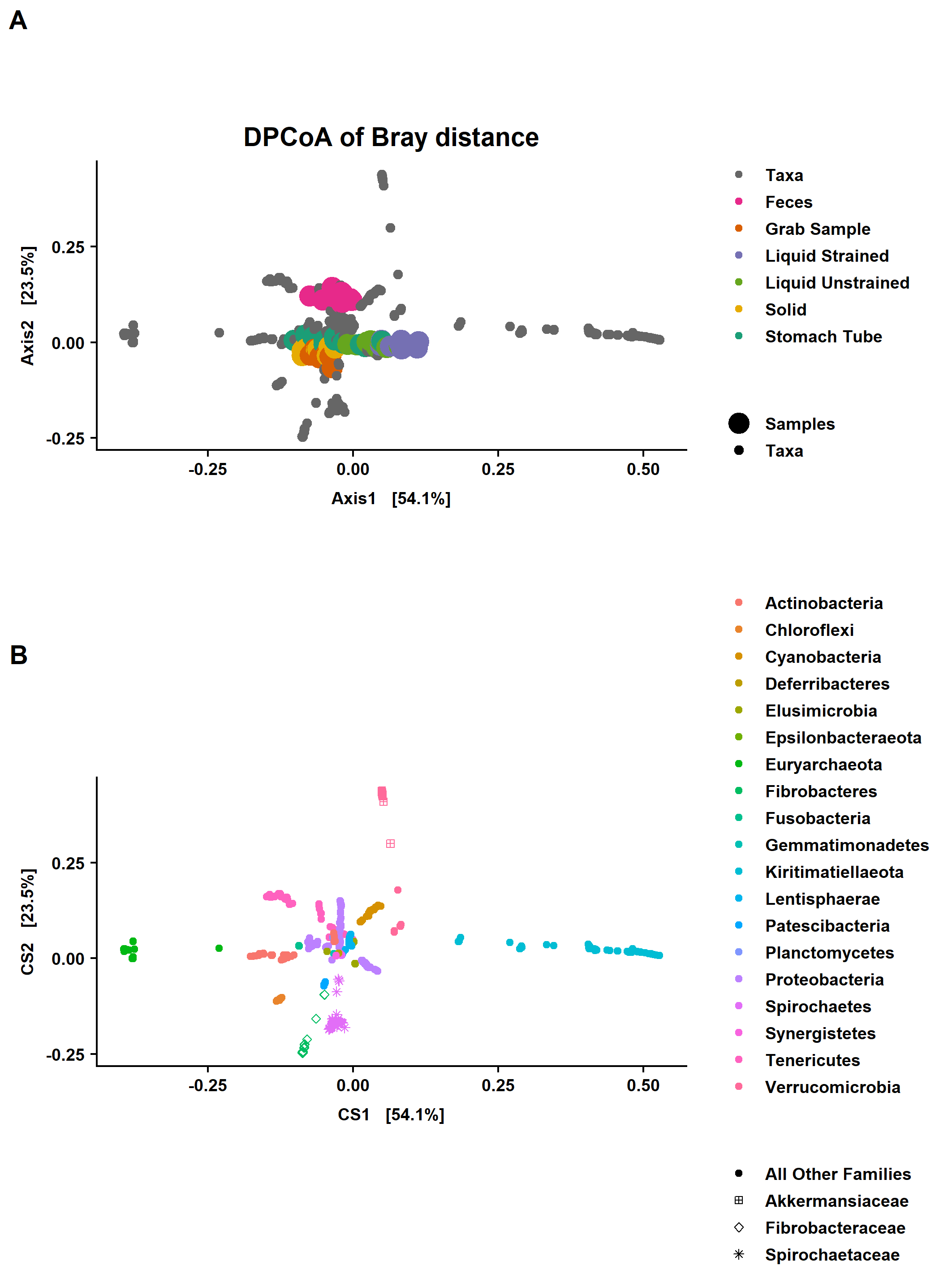

Supplement: S2 Fig — DPCoA is a phylogenetic ordination method and that provides a biplot representation of both (A) samples and (B) taxonomic categories. The 1st axis separtes liquid strained samples from other rumen sample types while the 2nd axis discrimates fecal from rumen samples. Samples that have larger scores on the 1st axis have more taxa from the phylum Kiritimatiellaeota and less taxa from the phylum Euryarchaeota. Likewise, samples with higher scores on the 2nd axis have more taxa from the family Akkermansiaceae and less taxa from the families Fibrobacteraceae and Spirochaetaceae. To faithfully reflect the variance in the coordinates, the height-to-width ratio was based on the ratio between the corresponding eigenvalues. (TIF) [file pone.0258176.s002.tif]

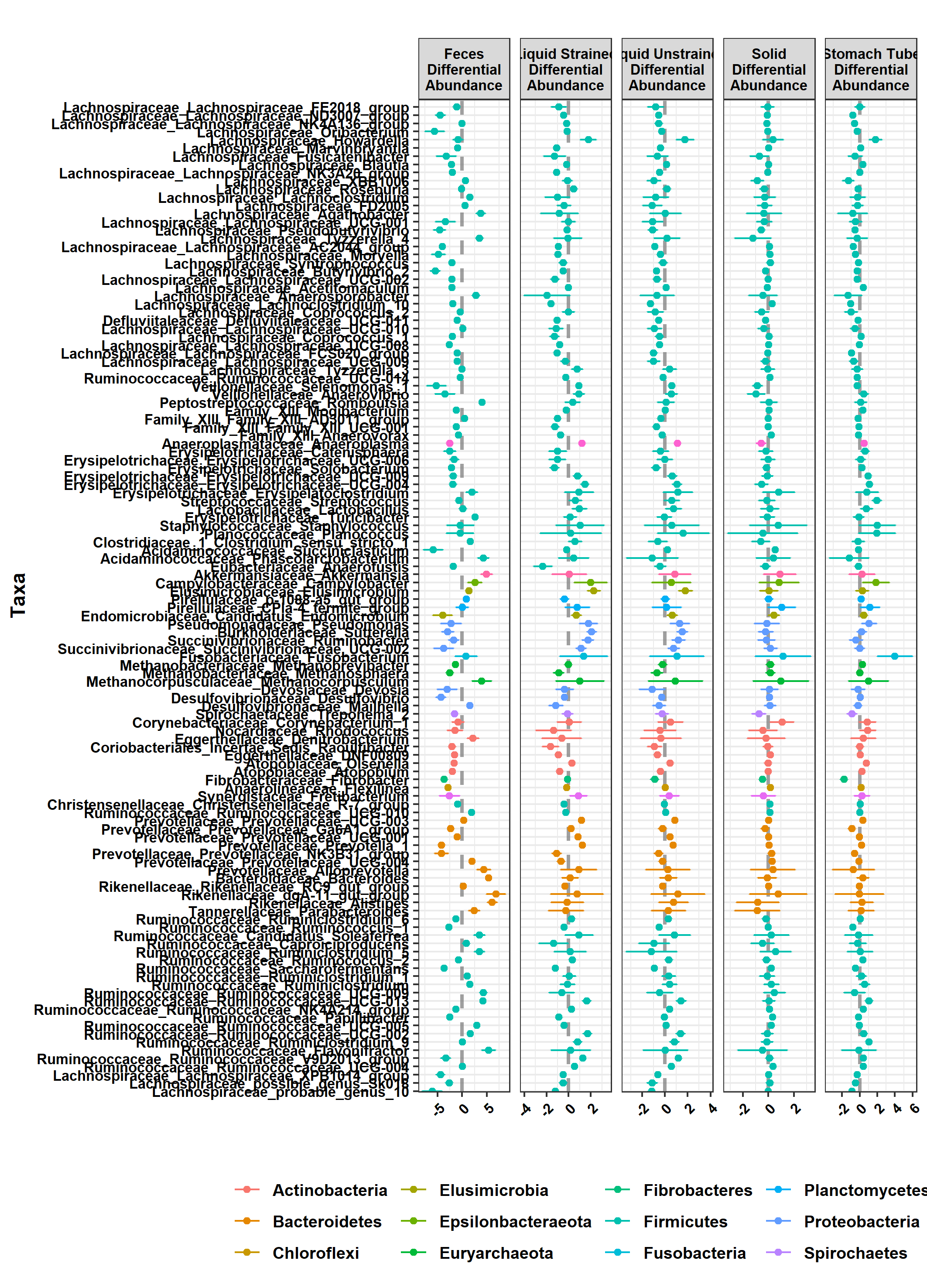

Supplement: S3 Fig — Taxa with negative coefficients for a sample type are expected to have a lower relative abundance when compared to the grab samples while positive coefficients suggest a higher relative abundance in that sample type compared to grab samples. Taxa are presented with phylum, family, genus and species to the lowest assigned level. (TIF) [file pone.0258176.s003.tif]
